# Parallel evolution of fluconazole resistance and tolerance in *Candida glabrata*

**DOI:** 10.3389/fcimb.2024.1456907

**Published:** 2024-09-27

**Authors:** Lijun Zheng, Yi Xu, Chen Wang, Yubo Dong, Liangsheng Guo

**Affiliations:** ^1^ Department of Ultrasound Medicine, The Second Affiliated Hospital of Soochow University, Suzhou, China; ^2^ Department of Pharmacy, The 960th Hospital of PLA, Jinan, China; ^3^ Department of Obstetrics and Gynecology, The Second Affiliated Hospital of Soochow University, Suzhou, China

**Keywords:** antifungal tolerance, antifungal resistance, *Candida glabrata*, calcineurin, Hsp90, evolution trajectory

## Abstract

**Introduction:**

With the growing population of immunocompromised individuals, opportunistic fungal pathogens pose a global health threat. *Candida* species, particularly *C. albicans* and non-albicans *Candida* species such as *C. glabrata*, are the most prevalent pathogenic fungi. Azoles, especially fluconazole, are widely used therapeutic options.

**Objective:**

This study investigates how *C. glabrata* adapts to fluconazole, with a focus on understanding the factors regulating fluconazole tolerance and its relationship to resistance.

**Methods:**

This study compared the factors regulating fluconazole tolerance between *C. albicans* and *C. glabrata*. We analyzed the impact of temperature on fluconazole tolerance, and requirement of calcineurin and Hsp90 for maintenance of fluconazole tolerance. We isolated colonies from edge, inside and outside of inhibition zone in disk diffusion assays. And we exposed *C. glabrata* strain to high concentrations of fluconazole and investigated the mutants for development of fluconazole resistance and tolerance.

**Results:**

We found temperature modulated tolerance in the opposite way in *C. albicans* strain YJB-T1891 and *C. glabrata* strain CG4. Calcineurin and Hsp90 were required for maintenance of fluconazole tolerance in both species. Colonies from inside and outside of inhibition zones did not exhibited mutated phenotype, but colonies isolated from edge of inhibition zone exhibited diverse phenotype changes. Moreover, we discovered that high concentrations (16-128 μg/mL) of fluconazole induce the simultaneous but parallel development of tolerance and resistance in *C. glabrata*, unlike the sole development of tolerance in *C. albicans*.

**Conclusion:**

This study highlights that while tolerance to fluconazole is a common response in *Candida* species, the specific molecular mechanisms and evolutionary pathways that lead to this response vary between species. Our findings emphasize the importance of understanding the regulation of fluconazole tolerance in different *Candida* species to develop effective therapeutic strategies.

## Introduction

The global landscape of invasive fungal infections has undergone a dramatic transformation over the past five decades, driven by the explosive growth of susceptible populations, including individuals with compromised immune systems, cancer patients, and those receiving aggressive chemotherapy or organ transplantation (Fisher et al., 2022). Invasive fungal infections caused by *Candida*, *Aspergillus*, *Cryptococcus*, and *Pneumocystis* species are the most commonly encountered (Malcolm and Chin-Hong, 2013). *Candida* species are the leading cause of invasive candidiasis, the most prevalent type of invasive fungal disease, and also rank among the top four causes of bloodstream infections, emphasizing the need for effective prevention and treatment strategies (Bassetti et al., 2017). While there are approximately 20 *Candida* species that can cause human disease, more than 90% of invasive candidiasis is attributed to just five species: *C. albicans*, *C. tropicalis*, *C. parapsilosis*, *C. krusei*, and *C. glabrata* (Pendleton et al., 2017). *C. albicans* and *C. glabrata* are the two most common pathogenic yeasts of humans (Azie et al., 2012), yet they are phylogenetically, genetically and phenotypically very different. The haploid *C. glabrata* is more closely related to the baker’s yeast *Saccharomyces cerevisiae* than to *C. albicans*, which predominantly exists as a diploid (Dujon et al., 2004).

Azoles are the most widely used antifungals, due to their accessibility, low toxicity, and broad spectrum of action. Azoles act by inhibiting lanosterol 14-α-demethylase, which converts lanosterol to ergosterol in fungus cellular membrane. Ergosterol, a fungal-exclusive sterol, is an essential component of the plasma membrane, where it maintains the membrane’s structural integrity and fluidity, enables the proper functioning of membrane-bound proteins, and facilitates the transmission of signaling molecules and various cellular processes (Jorda and Puig, 2020). The static nature and prolonged and extensive clinical use of azoles lead to the rapid emergence of azole resistance. The molecular mechanisms of azole resistance have been well characterized, and are generally attributed to two mechanisms: altered target and increased efflux (Lee et al., 2023). *C. albicans* has been the most extensively studied *Candida* species in relation to azole antifungal resistance. Point mutations and overexpression of *ERG11*, which encodes the target of azoles, as well as overexpression of drug efflux pump genes *CDR1*, *CDR2*, and *MDR1* are the most common mechanisms of azole resistance in both laboratory and clinical *C. albicans* strains (reviewed in (Whaley et al., 2016). *ERG11* does not appear to play an important role in clinical azole resistance in *C. glabrata* (Sanglard et al., 1999; Vermitsky and Edlind, 2004; Sanguinetti et al., 2005). The development of azole resistance in clinical isolates of *C. glabrata* is almost entirely attributed to the presence of activating mutations in the zinc cluster transcription factor gene *PDR1*, that lead to differential expression of downstream targets including the ABC transporters *CDR1*, *PDH1* (*CDR2*) and *SNQ2* (reviewed in (Whaley et al., 2016).

In addition to resistance, recent studies in *C. albicans* demonstrate tolerance is another distinct cellular response to antifungal drugs. Unlike resistance, which is defined as “the ability to grow in levels of drug that inhibit susceptible isolates”, antifungal tolerance is defined as “the ability of a subpopulation of cells from a susceptible isolate to grow, albeit slowly, in the presence of drug concentrations above established minimum inhibitory concentrations (MICs)” (Berman and Krysan, 2020). Antifungal resistance and antifungal tolerance are distinct phenomena. Antifungal resistance is typically caused by genetic or genomic mutations that directly affect drug-target interactions. Antifungal tolerance, on the other hand, is mediated by various stress response pathways, including heat shock responses, amino acid starvation responses, protein kinases such as protein kinase C, and epigenetic processes (Rosenberg et al., 2018; Berman and Krysan, 2020). Antifungal tolerance is also different from heteroresistance. Heteroresistance is a phenotype in which sub-population of cells exhibit increased resistance compared to the main population [Reviewed in (Yang and Berman, 2024)]. Recent studies from multiple research groups have consistently shown that the evolution of resistance and tolerance to the azole drug fluconazole is dose-dependent, with low doses promoting the development of resistance and high doses leading to the emergence of tolerance (Todd et al., 2023; Yang et al., 2023). Exposure to high concentrations of other azoles, such as posaconazole and miconazole, also induces tolerance, not resistance, in *C. albicans* (Kukurudz et al., 2022; Guo et al., 2024). Furthermore, low-dose fluconazole not only drives resistance but also triggers tolerance, although the frequency of mutations was significantly lower compared to high-dose treatment (Sun et al., 2023b). Tolerance has also been observed in other *Candida* species, such as *C. auris* (Rasouli Koohi et al., 2023) and *C. parapsilosis* (Sun et al., 2023a). However, the phenomenon and mechanisms of tolerance in *C. glabrata* remain understudied and unexplored.

Antifungal tolerance can be quantified using disk diffusion assays (DDAs), where tolerance is characterized by the presence of slow but continuous lawn growth within the zone of inhibition (ZOI), resulting from the microorganism’s ability to grow in the presence of supra-MIC concentrations of drugs (Rosenberg et al., 2018; Xu et al., 2021; Sun et al., 2023a, Sun et al., 2023b; Yang et al., 2023; Guo et al., 2024).

Azole tolerance has been extensively studied in *C. albicans* (Rosenberg et al., 2018; Xu et al., 2021; Sun et al., 2023a, Sun et al., 2023b; Yang et al., 2023; Guo et al., 2024). In contrast, there is a significant knowledge gap regarding azole tolerance in other *Candida* species, particularly *C. glabrata*, which is considered a distinct “unlike cousin” of *C. albicans* (Brunke and Hube, 2013).

The current study investigated both physiological and genetic factors regulating fluconazole tolerance in *C. glabrata*. We also investigated evolutionary trajectories in response to fluconazole challenge. We found both similarities and differences in factors and evolutionary trajectories between *C. albicans* and *C. glabrata*. This study demonstrates the value of comparative analysis in understanding the diversity of *Candida* species and their responses to antifungal agents and highlights the need for further investigation into the mechanisms underlying fluconazole tolerance in different *Candida* species.

## Materials and methods

### Strains and growth conditions

Two clinical isolates were used as wild-type strains: *C. glabrata* clinical isolate CG4 and *C. albicans* clinical isolate YJB-T1891. YJB-T1891 has been shown to be intrinsically tolerant to fluconazole (Yang et al., 2023). Stock cultures were preserved in 25% glycerol and stored at -80°C. Cells were grown on YPD-agar medium (1% yeast extract, 2% peptone, 2% D-glucose, and 2% agar) at the designated temperature. For growth in YPG medium, D-glucose was replaced with 20 g/L glycerol. The drugs were dissolved in dimethyl sulfoxide (DMSO) and stored at -20°C.

### Disk diffusion assay

Antifungal disk diffusion susceptibility testing was performed according to the CLSI M44-A2 guidelines (CLSI, 2009) with minor modifications. The strains were grown on YPD-agar plates and the cell density was adjusted to 1 × 10^6^ cells/mL. A 100 μL cell suspension was then plated onto each plate. A paper disk (GE Healthcare, USA) containing 200 μg of fluconazole was placed in the center of each plate. The plates were incubated for 24 hours at designated temperature and then photographed. Each strain was tested in triplicate, with three independent biological replicates.

To quantify tolerance, a custom R script called *diskImageR* was used, which calculates the radius of inhibition (RAD) to determine drug resistance and the fraction of growth (FoG) within the ZOI to determine tolerance. 20% drug inhibition (RAD_20_) and 20% growth within the ZOI (FoG_20_) are used as benchmarks to measure resistance and tolerance, respectively (Gerstein et al., 2016).

### Spot assay

Spot assay was performed as described previously (Xu et al., 2021). Briefly, Cell suspensions were prepared in distilled water and adjusted to a concentration of 1 × 10^7^ cells/mL. Three microliters of 10-fold serial dilutions were then spotted onto YPD agar plates with or without the addition of drugs. The plates were incubated at 30°C and photographed after 48 h. The experiment was repeated twice, with independent experiments conducted at distinct time points to ensure reproducibility.

For the detection of petite phenotypes, 3 μL of cells at a density of 1 × 10^7^ cells/mL were spotted onto YPD and YPG agar plates. The plates were incubated at 30°C and photographed after 24 h.

### Isolating colonies from disk diffusion assay plates

The CG4 strain was thawed from a -80°C freezer and streaked onto a YPD agar plate. The plate was incubated at either 30°C or 37°C for 24 hours to allow for colony formation. Colonies were then suspended in sterile distilled water and adjusted to a concentration of 5 × 10^4^ cells/mL. A 100 μL suspension was spread onto a new YPD agar plate. A paper disk containing 200 μg of fluconazole was placed in the center of the plate. The plate was incubated at the designated temperature for 36 hours to allow for cell growth. For the isolation of IZO and OZO colonies, a temperature of 30°C was used, while for EZO colonies, a temperature of 37°C was used.

### Obtaining fluconazole selected adaptors

This experiment was conducted following the approach we published previously (Zheng et al., 2023; Guo et al., 2024).The CG4 strain was thawed from a -80°C freezer and streaked onto a YPD agar plate. The plate was incubated at 37°C for 24 hours to allow for colony formation. The resulting colonies were then suspended in sterile distilled water and adjusted to a concentration of 1 × 10^7^ cells/mL. One hundred microliters of the cell suspension were spread onto YPD agar plates supplemented with fluconazole at concentrations of 8-128 μg/mL. The plates were incubated at 37°C for 3 days. From each plate containing fluconazole at concentrations of 16-128 μg/mL, 16 colonies were randomly selected.

### Data analysis

The images of DDA plates were analyzed using the *diskImageR* script (Gerstein et al., 2016). Statistical analysis was performed using one-way ANOVA test. A P-value < 0.05 was considered statistically significant.

## Results

### Temperature regulates fluconazole tolerance, not resistance, in *C. glabrata*

**Figure 1:**
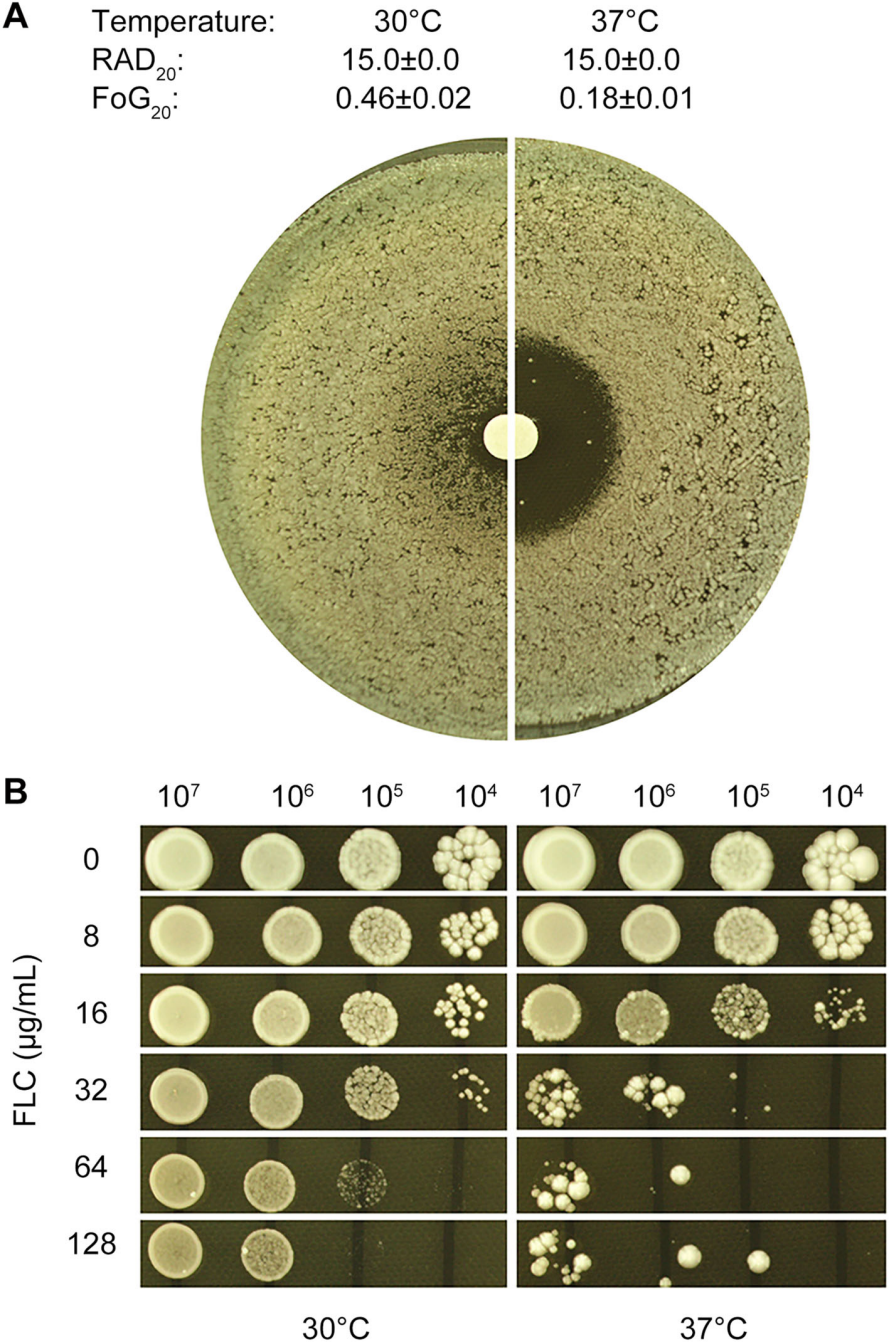
Temperature-dependent fluconazole tolerance in *C. glabrata* strain CG4 **(A)** Disk diffusion assay: 100 μL of cells at a density of 1x10^5^ cells/mL were spread on YPD-agar plates, and 200 μg fluconazole was applied to the disks. **(B)** Spot assay: 3 μL of 10-fold serial diluted cells were spotted on YPD-agar plates containing various concentrations of fluconazole, as shown in the figure. Plates were incubated at 30°C or 37°C for 48 hours **(A)** or 48 hours **(B)**, then photographed.

DDA plates were incubated at two distinct temperatures, 30°C and 37°C. At 30°C, inside ZOI, there was lawn growth, and colonies were noticeably smaller compared to those outside the ZOI. In contrast, at 37°C, the ZOI was clear, with only a few scattered colonies. Image analysis using *diskImageR* revealed that the strain exhibited the same RAD_20_ (15.0 ± 0.0) at both temperatures. However, the strain had FoG20 values of 0.46 ± 0.02 at 30°C and 0.18 ± 0.01 at 37°C. The FoG_20_ value was significantly higher at 30°C than at 37°C (p < 0.05, one-way ANOVA test) (**Figure 1A**). These findings suggest that temperature plays a modulating role in fluconazole tolerance but not resistance, with the test strain CG4 exhibiting greater tolerance at 30°C than at 37°C.

To assess the extent to which tolerance enhances the ability to grow in the presence of fluconazole, a spot assay was conducted. Cells grown at 30°C were able to grow in the presence of 128 μg/mL fluconazole, whereas cells grown at 37°C were significantly inhibited by 32 μg/mL fluconazole (**Figure 1B**). This suggests that tolerance plays a crucial role in allowing cells to survive and grow in the presence of fluconazole, with cells grown at 30°C exhibiting greater tolerance to the antifungal agent

### Fluconazole tolerance in *C. glabrata* is dependent on calcineurin and Hsp90

In fungi, Hsp90 and calcineurin are two essential regulatory proteins that play a critical role in responding to drug-induced stress. Hsp90, a vital molecular chaperone, regulates the structure and function of numerous key signal transducers, including calcineurin. Pharmacological inhibition of Hsp90 has been shown to prevent the development of azole resistance and reverse resistance in laboratory mutants and clinical isolates that have evolved resistance in a human host (Cowen and Lindquist, 2005; Cowen et al., 2006). Calcineurin, a calcium/calmodulin-dependent protein phosphatase, is a client protein of Hsp90, which stabilizes its protein. Calcineurin, in turn, confers resistance against azoles by activating the calcineurin-dependent pathway (Sanglard et al., 2003; Cowen, 2013).

**Figure 2:**
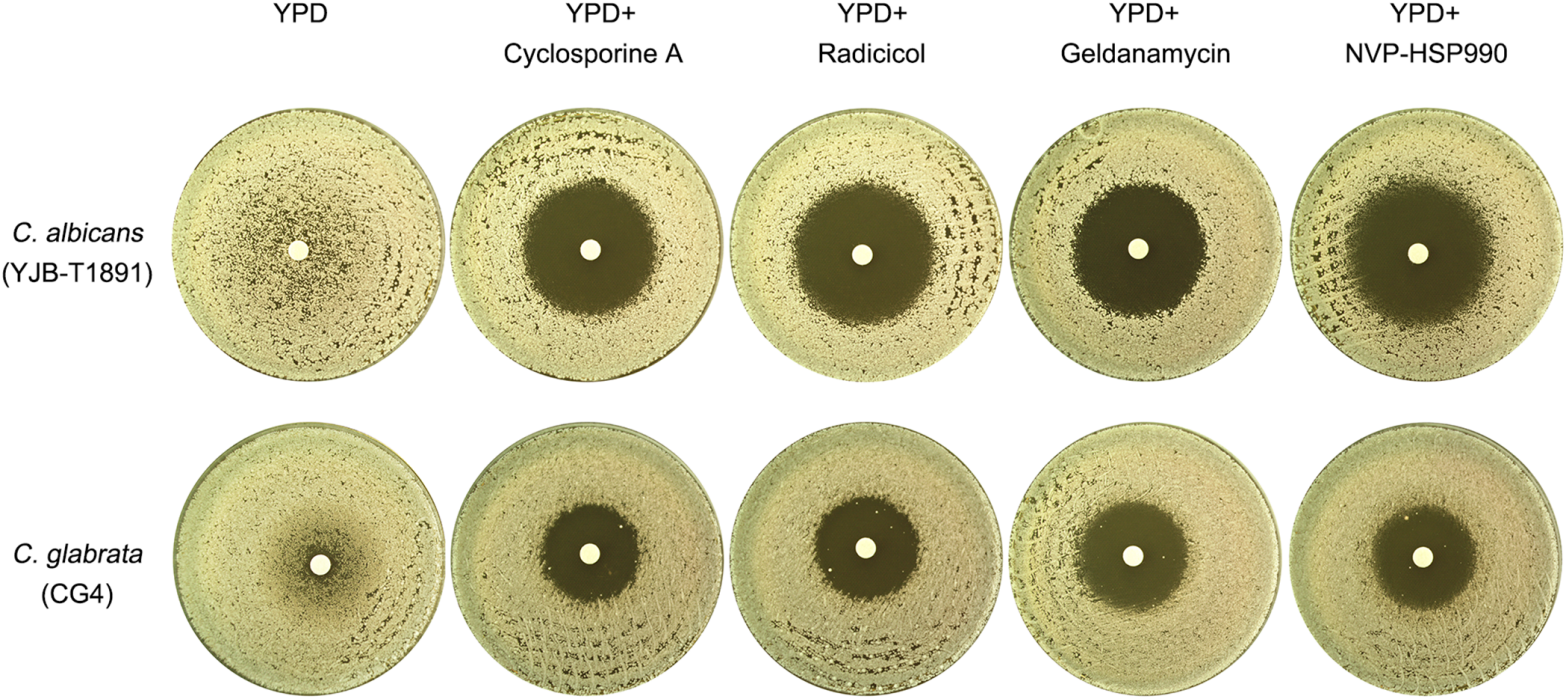
Comparative analysis of calcineurin and Hsp90 requirements for fluconazole tolerance in *C. albicans* and *C. glabrata* This figure compares the essentiality of calcineurin and Hsp90 for fluconazole tolerance in *C. albicans* and *C. glabrata*. Plates were supplemented with either calcineurin inhibitor cyclosporine A (0.5 μg/mL) or Hsp90 inhibitors radicicol (1 μg/mL), geldanamycin (10 μM) or NVP-HSP990 (10 μg/mL), as indicated in the figure. Fluconazole-containing disks (200 μg) were placed on the plates, which were then incubated at 30°C for 48 hours before being photographed.

In *C. albicans*, azole tolerance also relies on the cooperative actions of Hsp90 and calcineurin. The inhibition of Hsp90 by NVP-HSP990 or calcineurin by cyclosporine A abolishes tolerance to azoles, including fluconazole, ketoconazole, and miconazole (Rosenberg et al., 2018; Xu et al., 2021; Guo et al., 2024). This raises the question of whether tolerance to fluconazole is also dependent on Hsp90 and calcineurin in *C. glabrata*. We used the *C. albicans* tolerant strain YJB-T1891 as a control to investigate this. To test this, we cultured YJB-T1891 on YPD medium without adjuvants at 30°C. The resulting ZOI had a foggy appearance. The addition of cyclosporine A, a calcineurin inhibitor, resulted in a clear ZOI. Similarly, the addition 3 different inhibitors of Hsp90, radicicol, geldanamycin and NVP-HSP990, all resulted in a clear ZOI (**Figure 2**, top panel). Consistent with previous studies, our results demonstrate that tolerance to fluconazole in *C. albicans* is dependent on the cooperative actions of calcineurin and Hsp90.

To investigate the role of calcineurin and Hsp90 in fluconazole tolerance in *C. glabrata*, we tested CG4 on YPD medium at 30°C. In the absence of adjuvants, the strain exhibited a foggy ZOI. The addition of cyclosporine A, radicicol, geldanamycin or NVP-HSP990 resulted in a clear ZOI (**Figure 2**, bottom panel). Our results suggest that similar to *C. albicans*, fluconazole tolerance in *C. glabrata* is also dependent on calcineurin and Hsp90.

### Characterization of colonies within the zone of inhibition of tolerant strain

Yeasts can form petites which have deficiency in the aerobic respiratory chain. Petites for small anaerobic-sized colonies when grow in the presence of fermentable carbon source such as glucose, and are unable to grow on nonfermentable carbon sources such as glycerol (Day, 2013).

**Figure 3:**
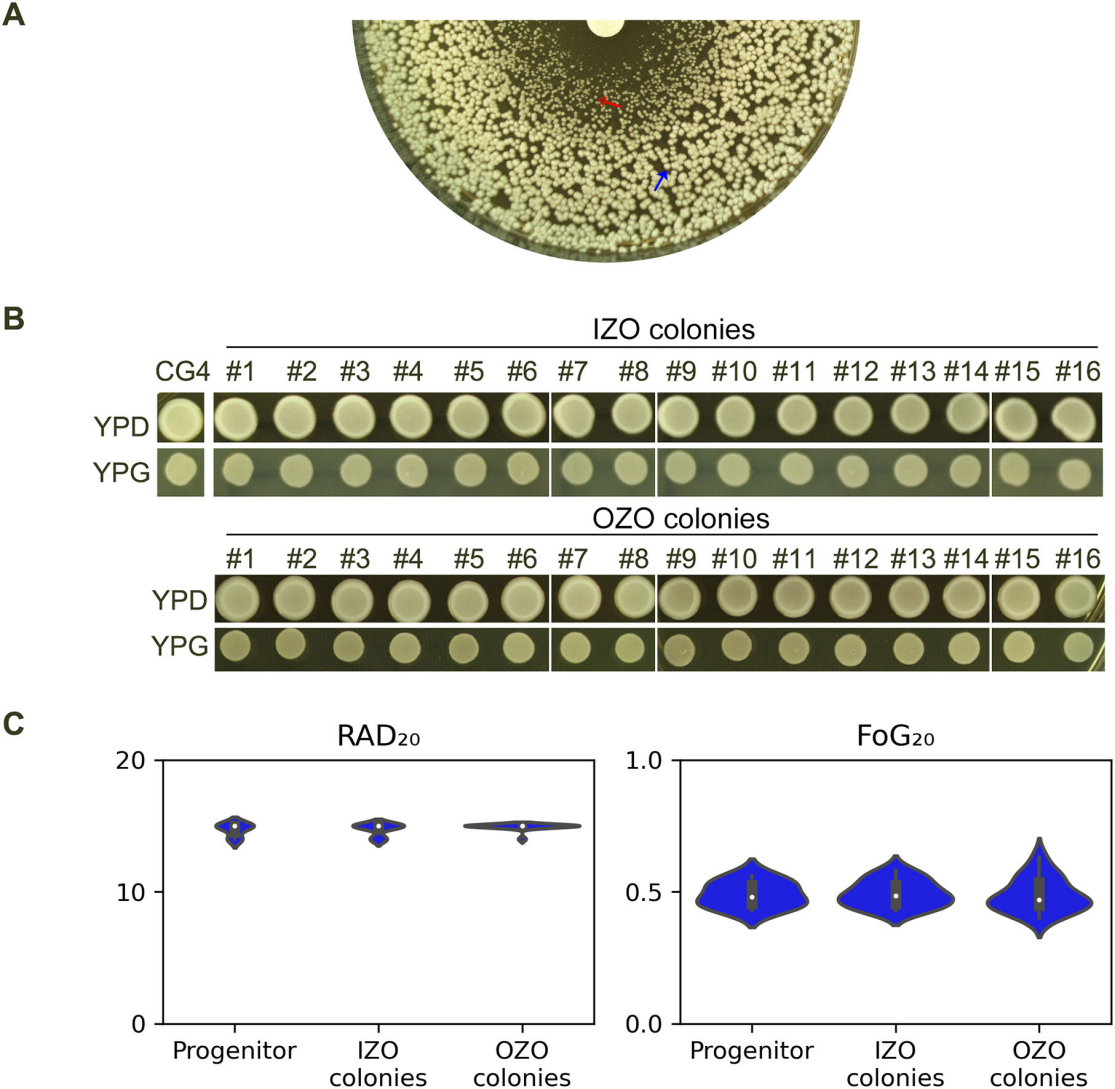
Carbon source utilization and fluconazole tolerance of CG4 colonies from inside and outside of zone of inhibition **(A)** Disk diffusion assay showing the zone of inhibition (ZOI) of fluconazole of CG4. IZO (red arrow) and OZO (blue arrow) colonies were randomly selected for further analysis. The plate was incubated at 30°C for 24 h to allow for optimal growth of the colonies. **(B)** Growth of IZO and OZO colonies on YPD and YPG plates containing glycerol as the sole carbon source. Colonies were suspended in distilled water and adjusted to 1.0x10^6^ cells/mL before spotting on the plates. The plates were incubated at 30°C for 24 h, after which the colonies were photographed. **(C)** Disk diffusion assay showing the tolerance of IZO and OZO colonies to fluconazole. The disks contained 200 μg of fluconazole, and the plates were incubated at 30°C for 24 (h) For the parent, 10 individual colonies were tested as 10 biological replicates. The images were analyzed using *diskImageR* script to quantify RAD_20_ and FoG_20_ values.

We investigated the nature of the colonies that form within ZOI of the strain CG4. As shown in **Figure 1**, CG4 exhibited lawn growth within ZOI at 30°C. The colonies from inside the zone of inhibition (**Figure 3A**, red arrow, hereafter referred to as IZO colonies) were significantly smaller than those from outside of the zone of inhibition (**Figure 3A**, blue arrow, hereafter referred to as OZO colonies), prompting us to investigate whether the IZO colonies were petites. 16 IZO and 16 OZO colonies were randomly selected and tested for their ability to grow on YPD and YPG plates containing glucose and glycerol, respectively. All colonies grew on YPG plates (**Figure 3B**). Furthermore, DDA was performed to assess the colonies’ tolerance. All colonies exhibited foggy ZOIs and had similar RAD_20_ and FoG_20_ values compared to the progenitor CG4 (**Figure 3C**), suggesting that the IZO colonies were not petites and both IZO and OZO colonies had similar tolerance to the progenitor.

### Colonies at the edge of ZOI have diverse phenotypes

**Figure 4:**
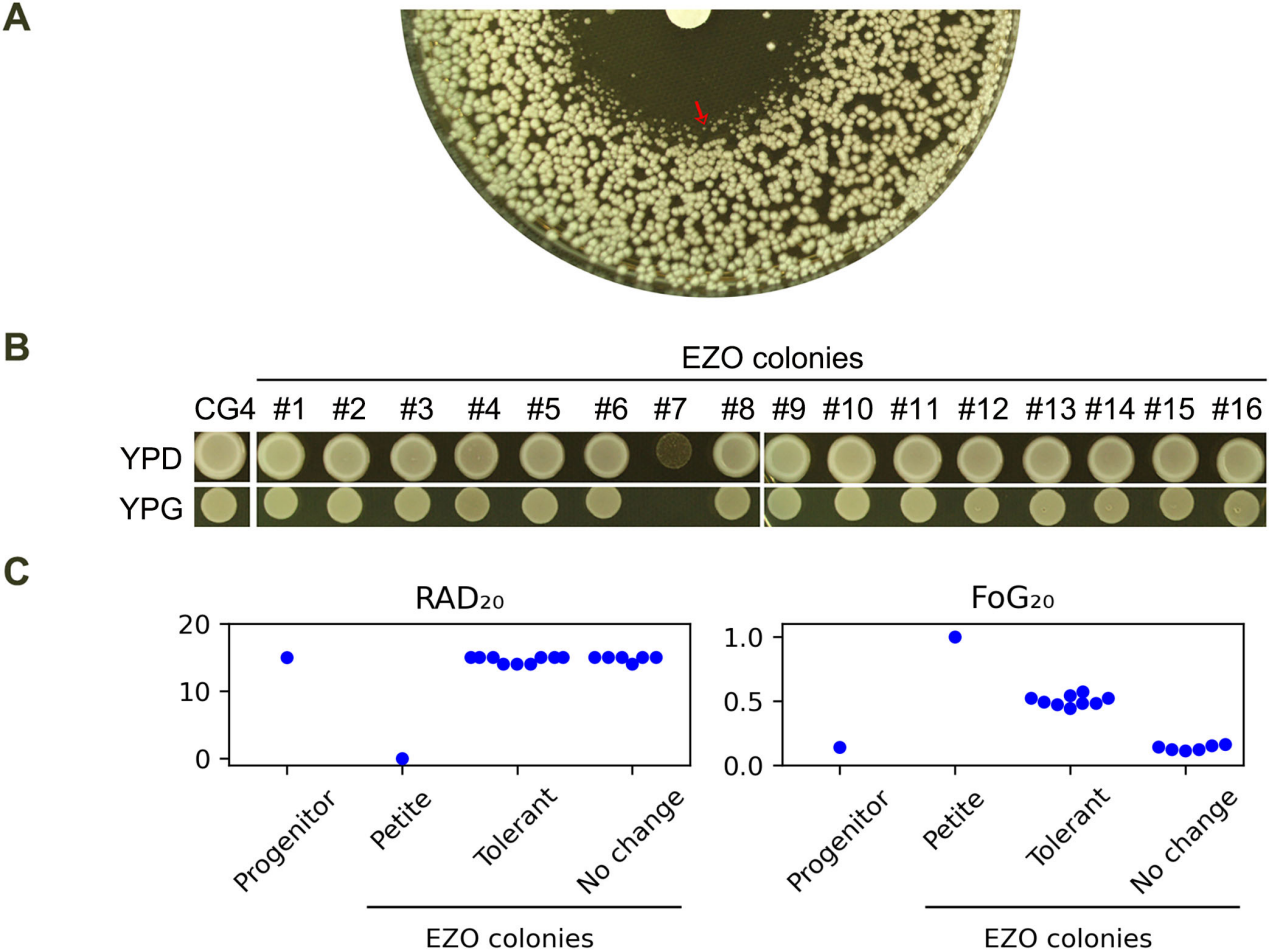
Characterization of EZO colonies **(A)** DDA of CG4 at 37°C, showing colonies at the edge of ZOI, (EZO colonies, red arrow) with reduced growth. **(B)** Growth of 16 randomly selected EZO colonies on YPG medium. Colony #7 failed to grow on YPG, indicating petite mutant phenotype. **(C)** DDA results of all 16 EZO colonies, with RAD_20_ and FoG_20_ values quantified using the *diskImageR* script. Mean values of 3 biological replicates are shown. Disks contained 200 μg fluconazole. Plates were incubated at 37°C for 24h and then photographed.

At 37°C, CG4 exhibited a clear ZOI against fluconazole. However, at the edge of the ZOI, colonies were noticeably smaller than those outside the ZOI (**Figure 4A**, red arrow). Sixteen colonies from this edge region (hereafter referred to as EZO colonies) were randomly selected, and one of them (#7) failed to grow on YPG plate, indicating it was a petite colony. The remaining 15 EZO colonies were able to grow on this plate (**Figure 4B**). When tested using a DDA, the petite colony exhibited no zone of inhibition, suggesting that it was highly resistant to fluconazole.

When tested at 37°C, CG4 exhibited a clear ZOI against fluconazole. However, colonies at the edge of the ZOI (hereafter referred to as EZO colonies) displayed significantly reduced growth, with smaller colony sizes compared to those outside the ZOI (**Figure 4A**, red arrow). A total of 16 EZO colonies were randomly selected for further analysis. One of these colonies (#7) was identified as a petite mutant, as it failed to grow on a YPG plate. In contrast, the remaining 15 EZO colonies grew normally on this medium (**Figure 4B**). DDA results revealed that the petite mutant (#7) exhibited complete resistance to fluconazole, as indicated by the absence of a ZOI. Among the 15 non-petite EZO colonies, 9 colonies (#1, #5, #6, #8, #9, #12, #14, #15, #16) displayed foggy ZOIs, suggesting tolerance to fluconazole. The remaining 6 non-petite colonies exhibited ZOIs similar to those of the progenitor CG4. DDA images were quantified using the *diskImageR* script. The progenitor CG4 had RAD_20_ and FoG_20_ values of 15.0 ± 0.0 and 0.14 ± 0.02, respectively. The 9 tolerant EZO colonies had RAD_20_ and FoG_20_ values of 15.0 ± 0.0 and 0.50 ± 0.04, respectively, whereas the 6 colonies without changes had RAD_20_ and FoG_20_ values of 15.0 ± 0.0 and 0.13 ± 0.02, respectively (**Figure 4C**). Thus, EZO colonies exhibited diverse phenotypes, including resistance and tolerance to fluconazole, highlight distinct evolutionary trajectories in *C. glabrata*.

### Exposure to high concentrations of fluconazole induces mainly resistance and occasionally tolerance

**Figure 5:**
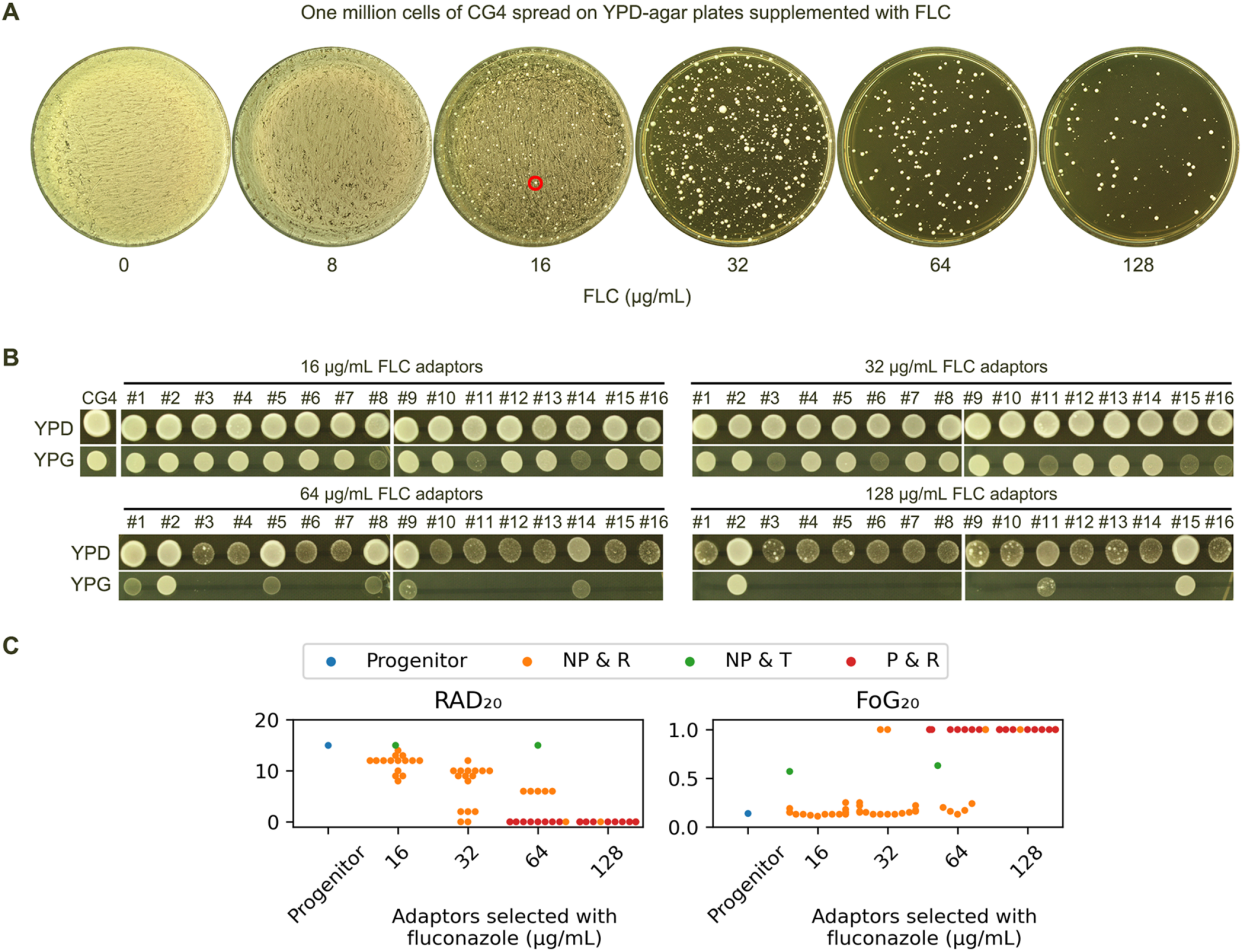
Fluconazole selects diverse resistant and tolerant adaptors **(A)** Approximately one million cells of CG4 were spread on YPD plates containing 8-128 μg/mL fluconazole. The plates were incubated at 37°C for 3 days, then photographed. The red circle indicates the colonies chosen for further analysis. **(B)** From each plate containing 16-128 μg/mL fluconazole, 16 random colonies (adaptors) were chosen. All 56 adaptors were spotted on YPD and YPG plates, incubated at 37°C for 24 h, and then photographed. **(C)** All 56 adaptors were tested with DDAs using disks containing 200 μg fluconazole. The results are presented as follows: P indicates petites, NP indicates non-petites, R indicates resistance to fluconazole, and T indicates tolerance to fluconazole. RAD_20_ and FoG_20_ values were quantified using the *diskImageR* script, and the mean of 3 biological replicates is shown for each strain.

Since CG4 was not tolerant to fluconazole at 37°C, we investigated how CG4 adapted to high concentrations of fluconazole. Approximately one million cells were spread on YPD plates containing 8-128 μg/mL fluconazole. The result showed that on the plate with 16 μg/mL fluconazole, although there was lawn growth, a few bright papillary colonies scattered on the plate (**Figure 5A**, red circle). Randomly, 16 such colonies (hereafter referred to as adaptors) were chosen for further analysis. On the plates containing 32-128 μg/mL fluconazole, a few hundred colonies appeared. Randomly, 16 colonies were chosen from each plate for further analysis. In total, we analyzed 56 adaptors.

On YPG plates, all adaptors selected by 16 and 32 μg/mL fluconazole could grow. However, 10 and 13 adaptors selected by 64 and 128 μg/mL fluconazole, respectively, failed to grow (**Figure 5B**). Combined with DDA results, the 56 adaptors were categorized into the following classes (**Figure 5C**):

Class 1 adaptors (n=23) were petites and resistant (P & R). They exhibited absence of ZOI on the plates, indicating they were highly resistant to fluconazole.

Class 2 adaptors (n=2) were non-petites and tolerant (NP & T). They were derived from 16 and 64 μg/mL fluconazole plates, respectively. Both adaptors had the same RAD_20_ as the progenitor. They had FoG_20_ values of 0.57 ± 0.09 and 0.63 ± 0.15, respectively.

Class 3 adaptors (n=41) were non-petites and resistant (NP & R). They had reduced RAD_20_ value as compared to the progenitor and clear ZOI.

Taken together, exposure to high concentrations of fluconazole selected mostly resistant adaptors and a few tolerant adaptors.

### Tolerance to fluconazole depends on calcineurin, while resistance does not

**Figure 6:**
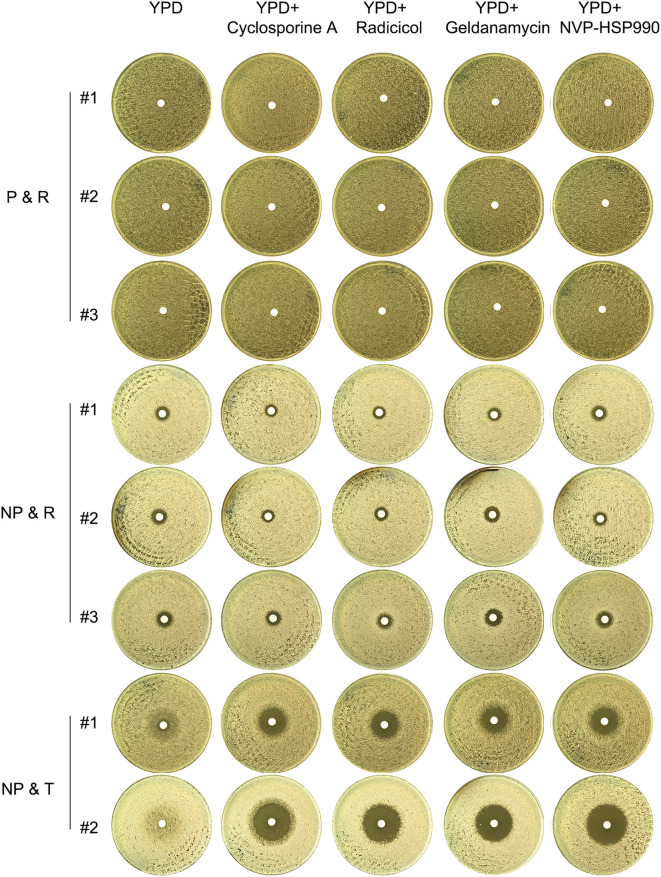
Requirement of calcineurin and Hsp90 for evolved resistance and tolerance Fluconazole-selected adaptors (P & R, NP & R, and NP & T) were tested with DDAs using YPD plates supplemented with calcineurin inhibitor cyclosporine A (1 μg/mL) or Hsp90 inhibitors radicicol (1 μg/mL), geldanamycin (10 μM) or NVP-HSP990 (10 μg/mL). The plates were incubated at 37°C for 48 hours and then photographed.

As shown in **Figure 2**, tolerance in the progenitor CG4 was dependent on calcineurin but not Hsp90. We investigated the dependency of the evolved tolerance and resistance on calcineurin and Hsp90. Randomly, 3 P & R colonies, 3 NP & R colonies and 2 NP & T colonies were tested with DDAs using YPD plates supplemented with cyclosporin A or NVP-HSP990 (**Figure 6**). The results showed that the P&R and NP&R adaptors exhibited similar ZOI on YPD plates with or without adjuvants, indicating that resistance in these strains was not dependent on calcineurin or Hsp90. In contrast, the NP&T adaptor displayed foggy ZOI on YPD and YPD with NVP-HSP990 plates, but clear ZOI on YPD with cyclosporine A. This suggests that tolerance in the NP & T adaptor was dependent on calcineurin, but not Hsp90.

## Discussion

The concept of antifungal tolerance, recently defined as the ability of fungal subpopulations to grow slowly in the presence of high concentrations of antifungal drugs, is best studied in *C. albicans*. Notably, tolerance and resistance are distinct phenomena with different molecular mechanisms and evolutionary trajectories, although the specific gene responsible for tolerance remains unknown (Sun et al., 2023b; Todd et al., 2023; Yang et al., 2023; Guo et al., 2024). Our study highlights the differences in the regulation of tolerance to azoles in *C. glabrata* compared to previous reports in *C. albicans.* Specifically, we found that tolerance in *C. glabrata* is differentially regulated by both physiological and genetic factors, whereas in *C. albicans*, the lab strain SC5314 was more tolerant at higher temperatures than at lower temperatures. In contrast, our results show that the test strain CG4 in *C. glabrata* was more tolerant at lower temperatures than at higher temperatures. Generally, resistance to fluconazole is attributed to altered targets and/or increased efflux. It remains unclear whether temperature influences fluconazole tolerance by regulating the genes involved in ergosterol biosynthesis and/or efflux. In *C. albicans*, strains with different genetic backgrounds exhibited varying levels of tolerance at 30°C and 37°C (Yang et al., 2023). It is essential to conduct more comparative studies using additional *C. glabrata* strains. These findings suggest that the evolutionary trajectory of antifungal tolerance may also differ between the two species. Specifically, our results indicate that in *C. glabrata*, high levels of fluconazole promote the simultaneous development of tolerance and resistance. Overall, our study highlights the importance of understanding the regulation of antifungal tolerance in different *Candida* species.

In both *C. glabrata* and *C. albicans*, resistance and tolerance evolve independently, and their emergence is not correlated. This implies that the mechanisms underlying resistance and tolerance are distinct and separate, and that they may be influenced by different factors. Notably, the extent of selective pressure drives the evolution of resistance and tolerance in *C. albicans*, with distinct outcomes resulting from different levels of pressure (Todd et al., 2023; Yang et al., 2023). In contrast, *C. glabrata* exhibits greater phenotypic variability under the same extent of selective pressure, with different cells adapting different evolutionary trajectories. This suggests that *C. glabrata* is more heterogenous than *C. albicans*, with a greater degree of genetic and phenotypic heterogeneity.

This study demonstrates that high concentrations of fluconazole select for three classes of adaptors: P & R, NP & R, and NP & T. Petite formation is a well-known mechanism of azole resistance in *C. glabrata* and its close relative *S. cerevisiae* (Whaley et al., 2016). Our results show that among the adaptors selected from plates containing 16-128 μg/mL fluconazole, the majority of those chosen from 64 and 128 μg/mL fluconazole plates were petites and resistant to fluconazole. However, none of the adaptors selected from plates containing 16 and 32 μg/mL fluconazole were petites, suggesting that the mechanism of azole resistance may vary depending on the concentration of the drug. Interestingly, petite formation may be a mechanism of azole resistance that is specific to very high concentrations of the drug.

Among the tolerant adaptors, one was selected by 16 μg/mL fluconazole, and one was selected by 64 μg/mL fluconazole. Similarly, in *C. albicans*, tolerance to fluconazole appeared on plates containing a wide range of concentrations of fluconazole. Thus, the extent of selective force does not appear to be the determinant of the frequency of appearance of tolerant mutations in both *C. glabrata* and *C. albicans*.

## Conclusion

Overall, our findings suggest that the evolutionary trajectory of antifungal tolerance differ between *C. glabrata* and *C. albicans*, and emphasize the importance of understanding the regulation of antifungal tolerance in different *Candida* species. Further research is needed to understand the molecular mechanisms underlying antifungal tolerance in *C. glabrata* and to identify the specific genes responsible for tolerance in this species.

## Data Availability

The raw data supporting the conclusions of this article will be made available by the authors, without undue reservation.
